# The up-rise in e-cigarette use – friend or foe?

**DOI:** 10.1186/s12931-016-0371-2

**Published:** 2016-05-17

**Authors:** Bo Lundbäck, Paraskevi Katsaounou, Jan Lötvall

**Affiliations:** Krefting Research Centre, Institute of Medicine, University of Gothenburg, SE-40530 Gothenburg, Sweden; Pulmonary and Critical Care Department, Evangelismos Hospital and School of Medicine, University of Athens, Athens, Greece

Following the millennium shift, the market for e-cigarettes has expanded logarithmically, and today there are hundreds of e-cigarette products and thousands of flavor variations. This increase in e-cigagette use contrasts to a major and continuing decrease in cigarette smoking seen in most westernized countries for some decades [1]. It seems that the marketing of e-cigarettes is less active in middle and low level income countries, as exemplified in a paper from Egypt in this issue of Respiratory Research [2].

Are e-cigs safe, or safe enough, is a controversy also in the medical society. Particularly the public health community seems to be divided, and safety and efficacy aspects of e-cigarettes for smoking cessation have been unclear. Among public health professionals, particularly in the UK [3], there is an opinion favoring the use of e-cigarettes as a smoking cessation tool among smokers, while others strongly believe the contrary [4]. So, who are those who use e-cigarettes? Is it middle aged or elderly smokers that want to quit smoking? According to the 2012 Eurobarometer survey, about 30 million adults in the 27 EU countries that year used or had used e-cigarettes [6], and the greatest proportion of ever users of e-cigarettes was found among subjects aged 15–24 years followed by those aged 25–39 years, further, this proportion was considerably greater among heavy smokers compared to light smokers.

And what about the usefulness of e-cigarettes in promoting smoking cessation, are they useful? A recently published meta-analysis and systematic review ended up in an opposite conclusion [7]. E-cigarette use was found to be associated with less quitting among smokers compared to quitting among smokers not having used e-cigarettes. The authors concluded that e-cigarettes should so far not be recommended for effective smoking cessation. In contrast to aiming at primary and secondary prevention of disease, subjects with already severe malignant smoking associated disease seem to be prone to switch to e-cigarettes, as demonstrated by one paper in this issue of Respiratory Research from the UK [8].

These controversies beclouding the effectiveness and safety of e-cigarettes serve as an important opportunity to reecho about the availability of very effective and safe drugs for the pharmaceutical treatment of nicotine addiction, which according to WHO, is a disease and should be treated accordingly. It is astonishing to find out that the two RCTs that were used to prove the effectiveness of e-cigarettes as a smoking cessation tool actually showed smoking abstinence (7 %) at 6 and 12 months (9–12 %) [9–11] lower than that of the placebo (10 %) in the main clinical trials done for the effectiveness of pharmaceutical therapy [12–15]. Abstinence with varenicline and bupropion in the same trials were 30 and 21 % at 6 months and 22 % and, 15 % at 12 months accordingly [14, 15]. In the UK, where e-cigarette use as a smoking cessation tool has been endorsed by the Royal College of Physicians of London ASH and NHS, there is currently an increasing use of e-cigarette as a smoking cessation tool and a decrease of the use of NRTs. Majority of health professionals are not trained in smoking cessation and hence have not adopted it in their everyday practice. Shouldn’t this be a concern that instead of health professionals going through training and conducting smoking cessation through scientifically endorsed best practices they engage in short cut advising on use of e-cigarettes for smoking cessation with no sufficient scientific backing?

Besides, the media’s widely quoted e-cigarettes being considerably less harmful to health as compared to conventional cigarettes [3–5] do not take into account long-term risks where we have no data. Although studies on the pulmonary effects of e-cigarettes are still limited, short-term negative effects of e-cigarettes are already emerging. As the airway epithelium provides the first line of defense against all inhaled exposures [16], e-cigarette use has lead to decreased exhaled NO and increased airway resistance in humans [24]. Moreover, the liquid flavors and chemicals used in e-cigarettes are in thousands with their detailed identification and effects being subject of research. Some early studies have found that levels of harmful constituents in e-cigarettes are lower than in ordinary cigarette smoke [17] and thus lead to the impression that e-cigarettes are unlikely to cause serious public health concerns, particularly relative to normal tobacco cigarettes, a statement made also by the authors of one publication in this issue of Respiratory Research [18], who also discuss ethical considerations of e-cigarette use for tobacco harm reduction. However, flavorings in e-cigarettes have cytotoxic effects in cell models [19], and another paper in this issue provides further evidence of pathological effects on the epithelium [20] with detrimental consequences for airway surface liquid homeostasis in habitual e-cigarette users [20]. In another study also presented in this issue several harmful chemicals including carcinogens were identified in e-cigarette vapour extract, which caused an increase in expression of CD11b and CD66b, and further, increased release of MMP-9. The authors conclude that pro-inflammatory responses from human neutrophils raise concerns over the safety of e-cigarette use [21], further e-cigarettes have an impact on respiratory flow resistance, impedance, and exhaled NO [22]. These are excellent examples of research about compositions, concentrations and effects of the e-cigarette liquids. Although the concentrations of nicotine in e-cigarettes are lower than in ordinary cigarettes, the use of e-cigarettes may result in nicotine dependence and thus also use of other tobacco products such as ordinary cigarettes [23]. As preventing young people from taking up smoking is a major public health issue, the focus of the marketing of e-cigarettes on young people raises severe concerns [23]. Further, inhaled nicotine in itself has probably health effects also on humans, as shown in animal models. Thus the recommended restriction or banning of e-cigarettes by the Forum of International Respiratory Societies (FIRS) is thus reasonable [24]. Hence we encourage the training of health professionals in smoking cessation and the implementation of the combination of approved pharmaceutical therapies and behavioral counseling in everyday practice until there is valid information available about the safety of e-cigarettes.
